# Deep Seated Lobular Capillary Hemangioma (Pyogenic Granuloma) of the Colon: A Rare Case Requiring Surgery beyond Endoscopic Management

**DOI:** 10.1155/2022/5641608

**Published:** 2022-03-29

**Authors:** Jaechun Park, Minjung Jung

**Affiliations:** ^1^Department of Radiology, Gospel Hospital, Kosin University, 262 Gamcheon-ro, Seo-gu, Busan, Republic of Korea 49267; ^2^Department of Pathology, Gospel Hospital, Kosin University, 262 Gamcheon-ro, Seo-gu, Busan, Republic of Korea 49267

## Abstract

**Background:**

Lobular capillary hemangiomas typically present as skin or oral mucosa lesions and have rarely been described in unusual sites, including the gastrointestinal tract. Most colonic lobular capillary hemangiomas, either asymptomatic or associated with GI bleeding, have been amenable to endoscopic treatment in literatures. *Case Presentation*. A 41-year-old woman presented with an incidental colonic mass during a systemic workup after adjuvant chemotherapy for HER2-positive breast cancer. Abdominal computed tomography revealed a deep seated colonic mass in the splenic flexure. An endoscopic strip biopsy was attempted for differential diagnosis of this lesion, but uncontrolled bleeding occurred, and an emergency surgery was eventually performed. Microscopic examination showed lobular capillary hemangioma involving full thickness of the colonic wall with mucosal ulceration.

**Conclusions:**

Colonic lobular capillary hemangioma is a benign vascular proliferation but is a candidate in differential diagnosis of benign or malignant tumors. Furthermore, the exceptional case may be deep seated and require more invasive surgery, unlike most cases of colonic lobular capillary hemangioma that can be treated with endoscopic modality.

## 1. Introduction

Lobular capillary hemangioma, also known as pyogenic granuloma, is benign vascular lesion and usually treated by excision. They typically present in skin or mucous membrane; however, some unusual cases have been reported in other anatomic locations, including the gastrointestinal mucosa. Herein, we report an exceptional case of colonic lobular capillary hemangioma involving full thickness of the colonic wall.

## 2. Case Presentation

A 41-year-old female underwent emergency surgery for bleeding after endoscopic strip biopsy. She had been diagnosed with HER2-positive breast cancer one year prior and underwent left breast-conserving surgery after treatment with neoadjuvant TCHP chemotherapy (docetaxel, carboplatin, trastuzumab, and pertuzumab). She received a systemic workup after completing eight cycles of adjuvant trastuzumab emtansine chemotherapy and intensity modulated radiation therapy, and an incidental colonic lesion was found.

**Figure 1:**
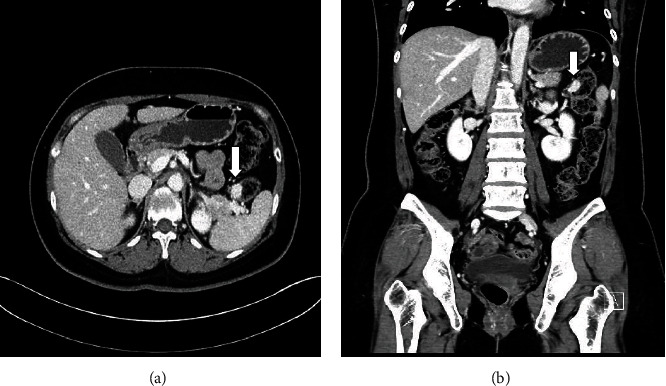
Radiologic findings. Contrast-enhanced CT image shows a 1.6 cm hypervascular tumor (arrow) in the splenic flexure: (a) transverse view and (b) coronal view.

The colonic lesion was observed as a 1.6 cm sized enhancing mass located in the splenic flexure on abdominal computed tomography (CT), although it had not been observed six months prior (Figure 1). It was confined to the colonic wall without extracolic extension, albeit unable to make a differentiation of the colonic wall layer, either an intramural subepithelial mass with an intraluminal growth pattern or transmural mass. It raised various radiologic differential diagnoses, including metastatic lesion of breast cancer, gastrointestinal stromal tumor, neuroendocrine tumors, neurogenic tumors, and vascular lesions, such as hemangioma or vascular aneurysm.

**Figure 2:**
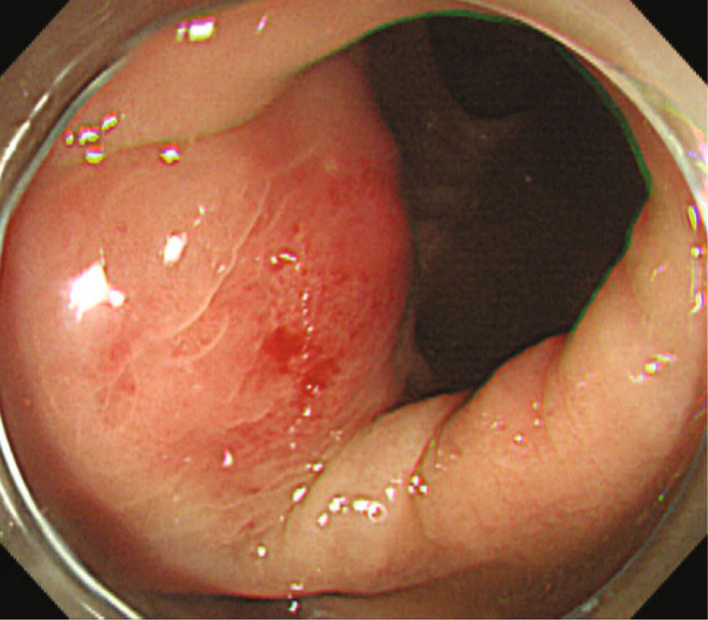
Colonoscopic finding. Nonulcerative, round, hyperemic lesion protrudes from the splenic flexure of the large intestine.

After six weeks, endoscopic evaluation was performed. The lesion was a round, hyperemic lesion protruding inward on colonoscopy, and a 2.1 cm sized intramural mass was observed on endoscopic ultrasonography (Figure 2). Two weeks later, strip biopsy was performed for tissue confirmation, and active bleeding occurred. Despite the use of a standard endoscopic hemostatic technique, bleeding was not controlled, and emergency hemicolectomy was performed.

**Figure 3:**
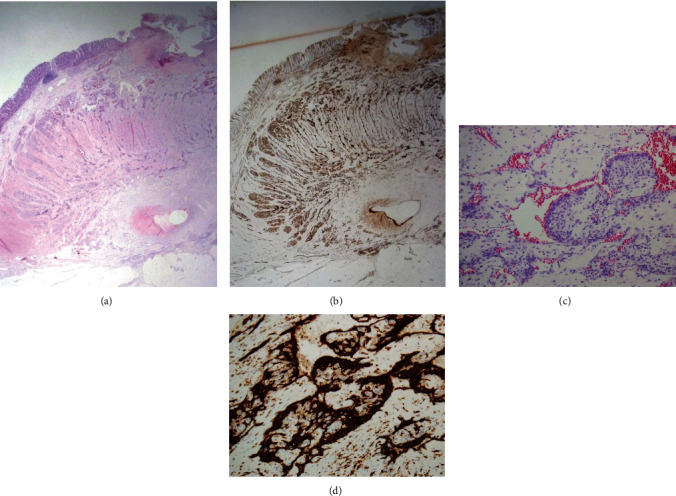
Pathologic findings. (a) Vascular proliferation is observed throughout the entire wall, centered on the muscularis propria (H&E staining, ×40). (b) In addition to the typical lobular arrangement, the feeding vessels exhibit vertical alignment (CD31 IHC, ×40). (c) Although most vascular endothelial cells are flat, the focal intraluminal protrusions are lined by cuboidal to columnar cells reminiscent of epithelial cells (H&E staining, ×100). (d) Immunostaining for CD31 confirms that the plump cells are endothelial cells (CD31 IHC, ×100).

Grossly, the lesion was a well-demarcated, fungating mass with mucosal ulceration measuring 2.2 cm in diameter. Microscopic examination revealed vascular proliferation in the muscularis propria with extension into the submucosa and pericolic adipose tissue. The overall vasculature was comprised of capillary vessels with a densely packed and lobular arrangement, and vertical alignment of the central vessels was prominent at the center of the lesion (Figures 3(a) and 3(b)). There were dilated vascular spaces and intraluminal blunt projections with collagenous cores, especially in the superficial portion adjacent to the ulcerative mucosa (Figure 3(c)). Most vascular endothelial cells were flat or protuberant. In contrast, the focal intraluminal projections were lined by cuboidal to columnar cells reminiscent of epithelial cells. Immunostainings CD34 and CD31 confirmed that these also were vascular endothelial cells (Figure 3(d)). The final pathologic diagnosis was lobular capillary hemangioma of the splenic flexure of the colon. The patient recovered well from surgery and completed 12 cycles of adjuvant trastuzumab emtansine chemotherapy.

## 3. Discussion

Lobular capillary hemangioma is a benign vascular lesion most commonly occurring at the skin, followed by the oral mucosa [1, 2]. However, this type of lesion has been reported in unusual sites, such as mucosa of the gastrointestinal tract, nose, larynx, eyes, and urogenital tract [3–8]. It is a rapidly growing mass, and clinical symptoms of bleeding, ulceration, and mass effects have been described.

Gastrointestinal lobular capillary hemangiomas also present with iron-deficiency anemia, melena, and hematochezia, as well as location-specific symptoms such as dysphagia, gastritis, intestinal intussusception, and tenesmus [3]. Considering the characteristics of hypervascularization and the superficial location of the gastrointestinal mucosa, it is expected that easy bleeding may occur. However, the largest case series conducted during 12 years at a single large-volume endoscopy center reported that most cases were asymptomatic, while a few experienced anemia (8%) and gastrointestinal bleeding (17%) [9]. The present case presented with an incidentally detected mass that grew during a short period of time and was asymptomatic. Bleeding was triggered by strip biopsy and was associated with iatrogenic mucosal ulceration.

Although the pathogenesis of lobular capillary hemangioma is not clear, various predisposing factors have been reported, such as injury, chronic irritation and hormonal changes [2, 10]. In addition, medications of oral contraceptives, systemic and topical retinoids, antiretrovirals, and antineoplastic drugs are implicated, and the latter include variable chemotherapeutic agents like epidermal growth factor-receptor (EGFR) inhibitors, EGFR tyrosine kinase inhibitors, tyrosine kinase inhibitors, BRAF inhibitors, inhibitors of MEK/ERK, vascular endothelial growth factor inhibitors, CD20 antagonists, and antimetabolites [11–14]. Given the rare frequency of gastrointestinal lobular capillary hemangioma, it is unclear whether the medications used such as chemotherapeutic agents in the presented case affect the pathogenesis. Nevertheless, gastrointestinal lobular capillary hemangioma suggests consideration of neoplastic conditions, especially in patients with malignant tumors, and might require tissue confirmation and therapeutic removal.

In gastrointestinal lobular capillary hemangioma, the most common treatment modality is endoscopic polypectomy or endoscopic mucosal resection [3]. These minimally invasive methods can be considered an appropriate choice given the relatively small size (median: 15 mm in diameter) and location of the mucosal epicenter [3, 9]. However, the presented case was a deep-seated lesion, for which surgical resection was an appropriate treatment approach. To the best of our knowledge, this is an exceptional finding that follows two other cases of deep-seated lobular capillary hemangioma in the English literature [15]. These prior cases were lobular capillary hemangioma that involved the entire thickness of the jejunum and the ileum, respectively, and they were relatively large (20 mm and 25 mm in diameter, respectively), similar to the size in the present case (22 mm).

## 4. Conclusion

We present a rare case of colonic lobular capillary hemangioma uniquely found in the deep layers. The latter makes invasive surgery more necessary than endoscopic treatment. In addition, it should be noted that the presence of a deep-seated location is not a sufficient finding to exclude lobular capillary hemangioma in the differential diagnosis of hypervascular colonic lesions.

## Data Availability

All data analysed during this study are included in this published article.
